# A Sorrow Shared Is a Sorrow Halved? Patient and Parental Anxiety Associated with Venipuncture in Children before and after Liver Transplantation

**DOI:** 10.3390/children8080691

**Published:** 2021-08-11

**Authors:** Antonia J. Kaluza, Anna Lisa Aydin, Berrit L. Cordes, Gianna Ebers, Albert Fuchs, Christiane Konietzny, Rolf van Dick, Ulrich Baumann

**Affiliations:** 1 Institute of Psychology, Goethe University, 60323 Frankfurt (Main), Germany; aydin@psych.uni-frankfurt.de (A.L.A.); berrit.cordes@stud.uni-frankfurt.de (B.L.C.); fuchs.albert@stud.uni-frankfurt.de (A.F.); van.dick@psych.uni-frankfurt.de (R.v.D.); 2 Department of Paediatric Liver, Kidney and Metabolic Diseases, Division of Paediatric Gastroenterology and Hepatology, Hannover Medical School, 30625 Hannover, Germany; Ebers.Gianna@mh-hannover.de (G.E.); Konietzny.Christiane@mh-hannover.de (C.K.); Baumann.U@mh-hannover.de (U.B.); 3 ERN TransplantChild, La Paz University Hospital, 28029 Madrid, Spain; 4 Institute of Immunology and Immunotherapy, University of Birmingham, Birmingham B15 2TT, UK

**Keywords:** liver transplantation, blood drawing, venipuncture, anxiety, pain, children, parents

## Abstract

Taking blood via venipuncture is part of the necessary surveillance before and after liver transplantation. The spectrum of response from children and their parents is variable, ranging from a short and limited aversion to paralyzing phobia. The aim of this retrospective, cross-sectional study was to determine the level of anxiety amongst children during venipuncture, to compare the anxiety reported by children and parents, and to identify the factors affecting the children’s and parents’ anxiety in order to develop therapeutic strategies. In total, 147 children (aged 0–17 years, 78 female) and their parents completed questionnaires. Statistical analysis was performed using qualitative and quantitative methods. Results showed that the majority of children reported anxiety and pain during venipuncture. Younger children had more anxiety (self-reported or assessed by parents). Children and parental reports of anxiety were highly correlated. However, the child’s anxiety was often reported as higher by parents than by the children themselves. The child’s general anxiety as well as the parents’ perceived stress from surgical interventions (but not the number of surgical interventions) prompted parental report of child anxiety. For children, the main stressors that correlated with anxiety and pain were factors during the blood collection itself (e.g., feeling the puncture, seeing the syringe). Parental anxiety was mainly related to circumstances before the blood collection (e.g., approaching the clinic, sitting in the waiting room). The main stressors mentioned by parents were the child’s discomfort and their inability to calm the child. Results indicate that the children’s fear of factors during the blood collection, along with the parents’ perceived stress and helplessness as well as their anticipatory anxiety are important starting points for facilitating the drawing of blood from children before and after liver transplantation, thereby supporting a better disease course in the future.

## 1. Introduction

Drawing blood via venipuncture is one of the most common, invasive procedures experienced by chronically ill patients [1]. This is particularly relevant in children who are liver transplant recipients and have blood drawn regularly as part of routine investigations both before and after transplantation. Even though it can be seen as a minor, invasive procedure [2], such repeated and frequent blood sampling is a stressful experience for young patients, often accompanied by pain and anxiety [3,4], which may impact long-term health, quality of life, and adherence to medical therapy.

Adverse experiences with blood collection and the lack of support in dealing with it do not only produce short-term negative effects on children, such as increased stress levels and increased anxiety [4,5], but can also have long-term negative consequences [6]: Firstly, it can decrease children’s cooperation during future venipuncture and also reduce their collaboration during future medical procedures more generally. Memories of pain can lead to avoidance of medical interventions [7] as well as medical noncompliance [8]. This may not only interfere with medical care but also result in a poorer disease course. Secondly, negative experiences of blood sampling can lead to a fear of needles and for some children, even needle phobia, which can persist into adulthood [6,9]. Especially intrusive medical procedures such as transplantation are often perceived as traumatic both by patients and their parents [10], increasing the risk of developing post-traumatic stress disorders among children [11]. Chronic childhood illnesses and organ transplantations are risk factors for the development of post-traumatic stress disorders (in adulthood) [10,11,12], and the perceived intensity of a medical treatment appears to be an important component of this risk [13].

However, the experience of fear, anxiety, and pain is highly individual and depends on various factors (e.g., age, gender, personality traits) [3,5,14]; little knowledge exists on blood sampling and anxiety among children before or after transplantation. This is remarkable since pediatric liver transplant recipients are frequently exposed to medical procedures due to their condition.

Furthermore, parental assessment and handling of their children’s anxiety as well as their own anxiety are also relevant. Several studies show that when parents are well-informed and support their children by using appropriate coping techniques during blood collection, the child’s anxiety can be reduced [4,15]. However, responding with appropriate behavior requires parents to correctly perceive and interpret their child’s anxiety. Such adequate response may not always be possible as parents may also suffer from anxiety and distress themselves [10]. Parents’ emotions and behavior are an important indicator for children as to whether a situation is frightening or not, thus having an important influence on the child’s own emotions. Correctly assessing a child’s anxiety and exhibiting appropriate behavior towards it can therefore reduce a child’s emotional stress [16,17]. At the same time, by taking an active role in the blood sampling process, parents themselves can increase their sense of control and thus reduce their own anxiety and distress [18], which in turn benefits the child.

Due to the far-reaching, negative, psychological, and physiological consequences brought about by the lack of attention to anxiety and pain during blood collection, it is important that chronically ill children learn to cope with blood collection and are offered adequate support. In order to develop suitable, therapeutic interventions for pediatric patients before and after liver transplantation, the aim of the present study was to determine the prevalence and level of anxiety among children, to compare the anxiety reported by children and parents, and to identify the factors that positively or negatively affect both the children’s and parents’ anxiety during blood collection.

## 2. Materials and Methods

### 2.1. Participants and Design

Children and their parents were recruited in a pediatric hepatology unit in Germany. Inclusion criteria was (planned) liver transplantation. All patients who have undergone or planned to undergo liver transplantation and were being monitored at our institution were formally invited with a personal letter providing the background and aims of this study. The final sample of participating children and their parents consisted of *N* = 147 participants (78 girls, 69 boys). The most frequent diagnoses were biliary atresia (*n* = 54), metabolic liver disease (*n* = 26), and acute liver failure (*n* = 11). The children were aged from 10 months to 17 years (mean (*M)* = 8.76, standard deviation (*SD)* = 4.83) and assigned to one of three age groups (0–3 years *n* = 23, 4–7 years *n* = 38, 8–18 years *n* = 86). In total, 126 participants had at least one surgical intervention (*M* = 3.51, *SD* = 2.86), 81 children underwent liver transplantation, 10–210 month (median 85 months) ago. For 66 children, transplantation was planned at the time of data collection. Blood samples were taken at an interval of 0–6 months in an inpatient or outpatient setting at the pediatric hepatology unit. Blood sampling was performed by medical and nursing staff familiar to the patients and their families.

### 2.2. Procedure and Materials

Questionnaires for children and their parents were constructed for this study; validated measures were applied and adapted to three different patient age groups. Children aged 4–7 years provided information on anxiety symptoms and pain as well as the perceived anxiety of their parents during venipuncture. Children aged 8–18 years provided additional information on stress caused by surgical interventions (not only transplantation), various stressors during blood-taking, and their trait anxiety. Parents provided information on stress caused by surgical interventions (not only transplantation), their own anxiety during venipuncture, various stressors during the process, the perceived anxiety and pain of their child during venipuncture, and the perceived trait anxiety of their child.

#### 2.2.1. State Anxiety during Venipuncture

Children rated their own and their parents’ state anxiety during venipuncture, with one item from the Children’s Fear Scale (CFS) [19,20]. We added a numeric scale (from 1 = *no fear at all* to 5 = *extreme fear*) to the five emoticons (neutral and fearful faces) to replace the verbal instructions in the original version. Parents assessed their children’s anxiety with an adapted version of the scale. Parental anxiety during venipuncture was assessed with the State-Trait Anxiety Inventory (STAI-SKD) [21], which measures state anxiety using five questions such as “I am concerned that something could go wrong” with the use of 4-point scales (from 1 = *not at all* to 4 = *very much*; α = 0.89).

#### 2.2.2. Trait Anxiety

Children’s trait anxiety was assessed using a self-report measurement (for children aged 8–18 years) and a parental-report measurement. The Spielberger’s State-Trait Anxiety Inventory for Children—Trait Version (STAIC-T) [22,23] is validated for children aged 8–18 years and assesses the occurrence of 20 self-reported anxiety symptoms within the past month (e.g., “I am afraid of making mistakes”; “I am worried about school”) using 3-point scales (1 = *hardly ever*, 2 = *sometimes*, 3 = *often*; α = 0.90). Parental report of the child’s trait anxiety was assessed with the STAIC-P-T [24], an adapted version of the STAIC-T by Strauss [25] containing 26 items, which mostly overlap with items from the STAIC-T (e.g., “He/she is afraid of making mistakes”; “He/she is worried about school”), also using 3-point scales (1 = *hardly ever*, 2 = *sometimes*, 3 = *often*; α = 0.90). As the STAIC-P-T (similar to the STAIC-T) was developed for parents of children aged 8–18 years, we used a shorter version containing 9 age-appropriate items for parents of children aged 4–7 years (α = 0.74) and one item (“During the last month, he/she was anxious”) for parents of children aged 0–3 years.

#### 2.2.3. Pain

We used the 6-point Wong–Baker FACES scale [26], which uses a combination of six emoticons (smiling and crying faces) and a numeric scale (from 0 = *no hurt* to 10 = *hurts worst*) to capture perceived pain in children (4–18 years). Parents assessed their children’s pain with an adapted version of the scale.

#### 2.2.4. Stress Caused by Surgical Intervention

Children aged 8–18 years were asked about their surgical intervention. If the child had more than one surgical intervention, we asked to refer to the most stressful one. More precisely, we asked (a) how bad the surgical intervention was for them, (b) how painful the surgical intervention was for them, and (c) how scared they were during the surgical intervention (α = 0.84). Furthermore, children were asked (d) how bad the surgical intervention was for their parents, and (e) how scared their parents were during the surgical intervention (item correlation *r*(74) = 0.88, *p* < 0.001). Parents of all children were asked (a) how bad the surgical intervention was for their child, (b) how painful the surgical intervention was for their child, and (c) how afraid their child was during the surgical intervention (α = 0.87). Furthermore, parents were asked (d) how stressful the surgical intervention was for themselves, and (e) how scared they were during the surgical intervention (item correlation *r*(132) = 0.67, *p* < 0.001). For all questions, a 5-point scale (from 1 = *not at all* to 5 = *extremely*) was used.

#### 2.2.5. Stressors during Venipuncture

Children aged 8–18 years rated ten stressors of the blood-drawing process (adapted from the Dental Fear Survey by Kleinknecht et al. [27]) and how much fear they experienced through them: (a) approaching the clinic, (b) sitting in the waiting room, (c) sitting in the treatment room, (d) smelling the disinfectants, (e) seeing the syringe, (f) searching for the vein, (g) feeling the puncture, (h) seeing the blood, (i) feeling the blood being drawn from the vein, (j) pressing the plaster onto the wound; a 5-point scale was used (from 1 = *no fear all* to 5 = *extreme fear*). Parents were asked how stressful it was for them (a) that their child was not feeling well during venipuncture, (b) that they could not calm their child during venipuncture, (c) that they were embarrassed by their child’s behavior during venipuncture, (d) seeing their child’s blood, and e) seeing their child being stung with a syringe; a 4-point scale was used (from 1 = *not at all* to 4 = *extremely*). The parents’ questions were developed by the authors of this paper in consultation with specialists in the field in order to capture different stressors for parents during the blood-taking process.

#### 2.2.6. Open Questions

We asked children (4–18 years) to tell us in their own words (a) what the worst was for them when the blood sample was taken, and (b) if there was anything that could help during venipuncture.

### 2.3. Data Analysis

Statistical analyses were performed using IBM SPSS version 27 (SPSS Inc., Chicago, IL, USA). In order to determine the prevalence and level of anxiety during venipuncture among children and their parents, we analyzed the frequencies of the children’s and parents’ ratings. In order to compare anxiety reported by children and parents, and to identify and quantify factors that affect children’s and parents’ anxiety during venipuncture, we used *t*-tests for dependent and independent samples, correlational analyses, repeated measures analysis of variance (ANOVA), and multiple regression analysis. The children’s open answers were coded and analyzed using the software MAXQDA 2020 for computer-aided qualitative text analysis [28]. We strictly followed the American Psychological Association’s guidelines in reporting all results, that is, we provided the sample distribution’s mean (*M*) and standard deviation (*SD*), the test statistics (*F* for ANOVA results or *r* for bivariate correlations), the degrees of freedom (in brackets, following the respective test statistic), and the level of significance (*p*-values and η^2^). For regression analyses, we provided the amount of variation explained (*R*^2^) and the regression coefficients (*b*) plus standard errors (*SE*) and confidence intervals (CIs).

## 3. Results

### 3.1. Preliminary Analyses

There were no gender differences regarding children’s anxiety during venipuncture, general anxiety, or pain—either from self-report data or from information provided by parents (see Table 1). Furthermore, parents did not report that surgical interventions were more stressful for their child or themselves depending on their child’s gender. However, girls (aged 8–18 years) reported significantly more stress caused by surgical intervention for themselves (*M* = 3.52, *SD* = 1.13) compared to boys (*M* = 2.56, *SD* = 1.17, *t*(62) = −3.33, *p* = 0.001). Girls also tended to report more stress caused by surgical intervention for their parents (*M* = 4.47, *SD* = 0.74) compared to boys (*M* = 3.98, *SD* = 1.24, *t*(49.21) = −1.97, *p* = 0.055). Both findings cannot be attributed to the number of surgical interventions or the duration of the illness.

Children’s ages were related to their anxiety during venipuncture and their perception of pain—both in the self-report data (*r*(121) = −0.47, *p* < 0.001; *r*(120) = −0.46, *p* < 0.001) and in the information provided by parents (*r*(141) = −0.45, *p* < 0.001; *r*(142) = −0.40, *p* < 0.001). Younger children reported more anxiety and pain (see Table 2 for means and standard deviations in the three age groups). Furthermore, the younger the child, the more stress was perceived by parents due to surgical intervention (*r*(132) = −0.29, *p* = 0.001) and the more anxiety parents reported for themselves during their child’s venipuncture (*r*(145) = −0.21, *p* = 0.013).

Children’s anxiety during venipuncture did not differ as to whether the transplant had already been performed (*M* = 2.29, *SD* = 1.37) or not (*M* = 2.46, *SD* = 1.42, *t*(121) = 0.68, *p* = 0.496).

Regarding children’s trait anxiety (independent from their venipuncture anxiety), we tested whether the children in our sample showed higher trait anxiety scores than children in a normalization sample (used to develop and validate the STAIC-P-T [24]). The children’s values (*M* = 40.85, *SD* = 9.32; 8–18 years, parent report) did not significantly differ from general anxiety values in a normalization sample (*M* = 41.28, *SD* = 8.46, *N* = 2953; *t*(3027) = 0.46, *p* = 0.643; see [24]). Even though no German standard values are available for self-reported trait anxiety in children, the mean values of our sample (*M*_female_ = 32.61, *M*_male_ = 31.43) were also comparable with the mean values obtained from the above-mentioned normalization sample (*M*_female_ = 34.76, *M*_male_ = 32.29). This means that the children in our sample, despite their stressful medical history, did not show higher general anxiety scores than children in a normalization sample, in either the self- or parent reports.

### 3.2. Prevalence and Level of Anxiety during Venipuncture—Children vs. Parent Report

Our results showed that the vast majority of children and parents experience anxiety and pain during venipuncture. In total, 89% of children aged 4–7 years, and 54% of children aged 8–18 years reported anxiety; furthermore, 92% of children aged 4–7 years, and 68% of children aged 8–18 years reported pain. According to their accompanying person (usually a parent), 61% of children aged 4–7 years, and 42% of children aged 8–18 years stated that they were afraid during venipuncture. With regard to the parents’ response, 78% of the parents of children aged 0–3 years stated that their child showed anxiety during venipuncture, and all parents in this age group stated that their child experienced pain. Regarding their own feelings, 73% of parents indicated that they were “tense”. Furthermore, parents reported a high level of state anxiety during venipuncture (*M* = 1.77, *SD* = 0.72), which was comparable to the state anxiety found in a validation study of participants in a threatening situation (*M* = 1.73., *SD* = 0.47, *N* = 40; *t*(185) = 0.01, *p* = 0.992; ([21]; Study 3)).

To compare levels of anxiety reported by children and their parents, we ran a 2 (Age Group (4–7 years, 8–18 years)) × 2 (Anxiety (parent rating, child rating)) ANOVA with repeated measures on anxiety. The analysis revealed a significant main effect for the age group (*F*(1,118) = 24.96, *p* < 0.001, η^2^ = 0.175) and a significant main effect for anxiety (*F*(1,118) = 26.52, *p* < 0.001, η^2^ = 0.183). The interaction between the two factors was not significant (*F*(1,118) = 0.58, *p* = 0.448, η^2^ = 0.005). This means that anxiety scores in children aged 4–7 years were higher (*M* = 3.43, *SD* = 0.20) compared to scores in children aged 8–18 years (*M* = 2.24, *SD =* 0.13), and parents reported higher anxiety scores for their children (*M* = 2.82, *SD* = 1.40) than the children did for themselves (*M* = 2.40, *SD =* 1.39), irrespective of the children’s ages (see Table 2 for means and standard deviations in all age groups).

Parent and children reports on the children’s anxiety during venipuncture were highly correlated (*r*(120) = 0.80, *p* < 0.001), and there was also a strong correlation between children and parent reports on the parents’ anxiety (*r*(124) = 0.54, *p* < 0.001). Furthermore, values of children’s general anxiety reported by children and parents were highly correlated (*r*(86) = 0.71, *p* < 0.001). This means that children and parents are evidently able to assess each other’s anxiety quite well. Nevertheless, the relationship between state anxiety scores also seems to be influenced by one’s own perception and possibly by projection as well: Upon examination of children’s anxiety reported by parents, there is a positive correlation with parents’ anxiety during venipuncture (i.e., the STAI-SKD score; *r*(143) = 0.40, *p* < 0.001), but when studying the children’s anxiety reported by the children themselves, the relationship with parents’ anxiety (self-report) weakens (*r*(123) = 0.17, *p* = 0.057). While the children’s anxiety is apparently not correlated with the (self-reported) anxiety of their parents, children do report more anxiety, depending on how much anxiety they *perceive* in their parents (*r*(123) = 0.30, *p* = 0.001).

### 3.3. Factors That Affect Anxiety during Venipuncture

To explore how different factors (i.e., general anxiety, number of surgical interventions, perceived stress by surgical interventions) affect children’s anxiety during venipuncture, we conducted multiple regression analyses. For parent report (all age groups, *N* = 147), the (z-standardized) independent variables explained a significant amount of variance in anxiety during venipuncture (*R*^2^ = 0.271, *p* < 0.001). As assumed, children with higher general anxiety showed more anxiety during venipuncture (*b* = 0.43, *SE* = 0.23, *p* = 0.001, 95% CI (0.19, 0.68)). Furthermore, perceived stress caused by surgical intervention (*b* = 0.56, *SE* = 0.13, *p* < 0.001, 95% CI (0.31, 0.81)), but not the number of surgical interventions (*b* = −0.07, *SE* = 0.04, *p* = 0.110, 95% CI (−0.15, 0.02)), predicted anxiety during venipuncture. For children’s report (8–18 years), the (z-standardized) independent variables also explained a significant amount of variance in anxiety during venipuncture (*R*^2^ = 0.303, *p* < 0.001). However, only the children’s general anxiety (*b* = 0.52, *SE* = 0.14, *p* < 0.001, 95% CI (0.25, 0.80)) was a significant predictor of their anxiety during venipuncture, but not the stress caused by surgical intervention (*b* = 0.20, *SE* = 0.14, *p* = 0.176, 95% CI (−0.09, 0.49)) nor the number of surgical interventions (*b* = −0.00, *SE* = 0.04, *p* = 0.951, 95% CI (−0.09, 0.09)), which may be due to the small sample size (*n* = 86).

### 3.4. Stressors during Venipuncture

Children aged 8–18 years felt frightened by different aspects of the blood-drawing situation: 66% by feeling the puncture, 59% by seeing the syringe, 48% by the staff searching for the vein, 47% by feeling the blood being drawn from the vein, 45% by sitting in the treatment room, 33% by approaching the clinic, 29% by seeing the blood, 28% by sitting in the waiting room, 16% by smelling the disinfectants, and 11% by pressing the plaster onto the wound. All stressors were moderately or highly related with anxiety and pain during venipuncture, the exception being “smelling the disinfectants”, which did not yield (highly) significant correlations, and “pressing the plaster onto the wound”, with only small to moderate correlations. With regard to anxiety, the highest correlations were found for “seeing the syringe” (children self-report: *r*(83) = 0.79, *p* < 0.001; parent report for children: *r*(82) = 0.78, *p* < 0.001). With regard to pain, correlations were the highest for “feeling the puncture” (children self-report: *r*(81) = 0.74, *p* < 0.001; parent report for children: *r*(81) = 0.53, *p* < 0.001). Interestingly, those aspects did not play a role in relation to parents’ anxiety, which was only related to stressors for children *before* the actual venipuncture, namely, approaching the clinic (*r*(84) = 0.26, *p* = 0.017), sitting in the waiting room (*r*(84) = 0.30, *p* = 0.005), and sitting in the treatment room (*r*(84) = 0.33, *p* = 0.002).

Parents reported that they were negatively affected by different stressors during venipuncture: 81% by their child not feeling well during venipuncture, 56% by seeing their child being stung with a syringe, 50% by not being able to calm their child during venipuncture, 19% by seeing their child’s blood, and 11% by being embarrassed about their child’s behavior during venipuncture. Two of these five potential stressors were related to the anxiety felt by both parents and children: a) the more parents felt stressed about their child not feeling well, and b) the more they felt stressed about not being able to calm their child, the higher the anxiety scores were for their children (children self-report: (a) *r*(122) = 0.40, *p* < 0.001; (b) *r*(122) = 0.38, *p* < 0.001; parent report for children: (a) *r*(140) = 0.50, *p* < 0.001; (b) *r*(140) = 0.53, *p* < 0.001); and for themselves: (a) *r*(144) = 0.58, *p* < 0.001; (b) *r*(144) = 0.42, *p* < 0.001). The same pattern of results was found for the relationship between the two stressors and pain (children’s self-report: (a) *r*(119) = 0.37, *p* < 0.001; (b) *r*(119) = 0.38, *p* < 0.001; parent’s report for children: (a) *r*(139) = 0.43, *p* < 0.001; (b) *r*(139) = 0.37, *p* < 0.001).

Regarding the duration of the children’s illness, we also found significant correlations between children’s perceived pain (*r*(108) = −0.23, *p* = 0.016), children’s anxiety (*r*(109) = −0.28, *p* = 0.003), and parents’ reports of their children’s anxiety (*r*(125) = −0.30, *p* = 0.001). This means that the longer the children were ill, the less pain and anxiety they reported. 

Finally, we analyzed the children’s answers to the open questions: (a) what was the worst for them when the blood sample was taken, and (b) if there was anything that could help during venipuncture. In the following, we report the three most common answers to those two questions. Of 124 children aged 4–18 years, 43 children named the insertion of the needle as the worst, 20 children mentioned the search for the vein, and 16 children answered that being held in place by someone was the worst. As for the helpful factors, 33 children mentioned the presence of their parents, 18 children named distraction, and 8 children answered that a gift from a toy box could help them.

## 4. Discussion

The aim of the present study was to examine anxiety associated with venipuncture in children with liver disease before or after liver transplantation. Our results indicate that the majority of children reported anxiety and pain during venipuncture, which was consistently reported by both children and parents. This was the case for children before or after liver transplantation. Parents’ and children’s perception of anxiety were strongly correlated; however, on average, parents rated their children’s anxiety higher than the children themselves. Factors which were related to children’s anxiety during venipuncture were the child’s age, their general trait anxiety, and aspects of the blood collection itself, e.g., the insertion of the needle. Parental anxiety, on the other hand, was more associated with aspects before the actual venipuncture (e.g., approaching the clinic with an anxious child), that is, anticipatory anxiety played a greater role for them. The main stressors mentioned by parents were the lack of options for action (e.g., not being able to calm the child) and the discomfort of the child. There was also evidence that children’s anxiety during venipuncture is related to the stress induced by former surgical interventions (e.g., transplantation); however, this finding depended on the source of information (i.e., parental report) and requires further validation in a larger sample.

Our findings also indicate that parents similarly experience anxiety during blood collection, which was perceived by the children. This finding is important as parental anxiety and their distress symptoms could influence their children’s threat perception, as argued by Stuber and Shemesh [10], and thus the children’s anxiety [29]. This can also be seen as a form of vicarious learning from the observation of others.

Comparing the anxiety reported by children and parents revealed that parents reported higher anxiety levels in their children than the children themselves. Furthermore, the relationship between children’s and parents’ anxiety during venipuncture was higher when observing their own fear and the *perceived* fear of the other party, than the *actual* fear (i.e., self-report) of the other party. This is in line with findings reported by Krain and Kendall [30] and indicates that this overestimation might have an unfavorable effect on children’s anxiety (via feedback processes); therefore, parents should be involved in possible therapeutic interventions. This is particularly important, since the anxiety of both children and parents seems to be influenced by their *perception* of the other’s anxiety rather than their *actual* (albeit also self-reported) anxiety.

Different factors were related to the children’s anxiety. While previous research provides mixed results regarding the role of gender [2,29,31], our results revealed that children’s gender had no effect on their anxiety and pain. However, we did find significant differences for age. Younger children, in particular, suffer from greater anxiety and pain, which is consistent with previous research [2,3,5,32]. In addition, parents of younger children also reported more anxiety during blood collection and a greater stress level resulting from surgical interventions. This could be due to the fact that blood collection from younger children and infants is often more difficult to perform, is taken from venipuncture sites that appear threatening to non-professionals (e.g., blood collection from the head), and coping options (e.g., distraction) are generally limited.

Even though the children in our sample did not show increased general anxiety levels (compared to healthy controls in a normal sample), trait anxiety was a significant predictor of children’s anxiety during venipuncture. This indicates that children with higher general anxiety (in the sense of a personality trait) have an increased need for support with regard to anxiety during blood collection.

Another factor predicting children’s anxiety was the perceived stress of previous surgical interventions (reported by parents), but not the number of interventions. This is in line with previous findings, which reported that the number of blood collections or medical interventions is not relevant or even leads to less anxiety [31,33]. Our findings thus highlight that it is not the objective risk that is critical in the development of anxiety and posttraumatic symptoms, but the subjective assessment of the medical procedure as stressful and painful [10]. This points to an important starting point for practical interventions, for example, by providing parents and children with better information on surgical procedures, thus strengthening their sense of control. Future research may examine whether disease burden at the time of transplantation (or other surgical interventions) is associated with perceived stress caused by medical intervention. This information could disentangle possible effects, as recent research has shown that various markers of illness severity around transplantation can impair patient outcome such as cognitive functioning [34].

Furthermore, the results on stressors during venipuncture highlight that both aspects of the blood collection itself (including seeing the syringe, feeling the puncture) and factors before blood collection (e.g., approaching the clinic) provoke anxiety. Interestingly, those aspects affect children’s and parents’ anxiety during venipuncture differently: Whereas children perceive the actual process of drawing blood as the most frightening, which is also reflected in their responses to the open questions, parental distress is rather associated with their child’s anticipatory anxiety before the actual venipuncture. Thus, these results indicate that interventions should not only address acute anxiety during blood collection but should also focus on anticipatory anxiety. In addition, the parents’ concern that their child was not feeling well, or that they could not calm their child was also a predictor of the child’s anxiety and of the parents’ own anxiety. As argued by Stuber and Shemesh [10], parental functioning is very important for chronically ill children. Although children in our sample indicated parental presence as the most helpful factor (as reflected in their answers to the open questions), certain parental behaviors may exacerbate rather than reduce children’s anxiety [16,17,33]. Therefore, it seems important that parents actively support their children—for example, by distracting them—rather than just passively watch during venipuncture [35].

This study has several limitations. Firstly, we collected data from only one hospital in Germany, which may limit generalizability. A larger sample from different hospitals in different countries is necessary to evaluate the generalizability of our findings. A larger sample could then also look into different age groups (such as children above and below 14 years of age). Secondly, the cross-sectional nature of the data limits conclusions about causality. It is possible, and even likely, that the relationships are bidirectional and that the different factors influence each other. For example, the relationships between the anxiety scores of children and parents may indicate not only that children’s anxiety is influenced by parental anxiety, but also that parental anxiety is influenced by children’s anxiety. Future research might address this question of causality by using longitudinal studies with fully lagged designs. Thirdly, children and parents participated voluntarily. This may have introduced bias into the sample. For example, those patients for whom blood collection was very stressful may not have completed the survey because they preferred to avoid the topic. Finally, our research relies on subjective data, i.e., information provided by pediatric patients and their parents. Objective measures of stress and anxiety, e.g., adrenalin and cortisol release, do not always correspond with self-reported data [36]. One reason for this discrepancy in psychological and physiological stress reactions may be the bias in self-reporting, which can be influenced by the social desirability of the respondents as well as by self-protective or strategic concerns [37]. However, due to the survey’s anonymous nature (the participating children filled in the questionnaire independently and anonymously) and the fact that the subjective perception of fear and pain are the most relevant in suffering and therapeutic intervention, this limitation seems rather negligible.

Besides the above avenues for future research, we also suggest to the inclusion of further concepts, namely, anxiety sensitivity (AS) and catastrophic interpretations. Several studies have shown that AS is significantly correlated with fear of pain, and studies of nonclinical pain have provided further support for a link between AS and pain. Models of the association between AS and pain emphasize the importance of the fear of pain in worsening the experience of pain. These models suggest that an elevated AS amplifies the fear of pain, thus leading to avoidance and a subsequent increase in pain [38,39]. Measuring and controlling for AS could hence be useful in future research. Catastrophic interpretations may also have an impact on anxiety and pain during blood collection. Pain catastrophizing, for instance, is an exaggerated negative appraisal when facing (potential) pain, with the patient expecting the worst possible outcome in an irrational way [40,41]. Future research may look at extreme groups of children and see if catastrophizing may be related to their anxiety and pain interpretations.

The results of this study point to important practical interventions. Firstly, they highlight the prevalence of anxiety in children with chronic diseases during blood collection and therefore emphasize the need for intervention. Previous research has focused primarily on intervention in children and has demonstrated that psychological intervention [2], distraction [35,42] as well as local anesthetics [43] can reduce anxiety during blood collection. Secondly, our results also point to two other starting points: parental anxiety as well as anxiety about blood collection, namely, anticipatory anxiety. Parents should be informed about and be actively involved in the blood collection, as this will help them to better support their children and can reduce the child’s anxiety [15]. This could be done, for example, through psychoeducational material prior to the blood collection [44]. The child could also be fully informed about the blood collection in advance, which would increase their sense of control and thus reduce anxiety during the blood collection [45,46]. Another starting point for reducing the anxiety of both parents and children would be relaxation techniques, which could be performed both before blood collection and during the procedure [47,48].

## 5. Conclusions

In summary, our study offers a meaningful contribution to the treatment of children with chronic diseases. The results indicate that children’s fear of aspects of the blood collection itself, as along with their parents’ perceived stress and helplessness as well as their anticipatory anxiety are important starting points for facilitating venipuncture in children with liver transplantation in the future. Finding ways to manage such conditions would thus increase cooperation with treatment and support a better disease course. It is important to note that it is often not the objective situation (stressor) but more the psychological appraisal (interpretation) of this situation that affects behavioral and emotional responses. We believe that these factors should thus be taken seriously. Based on the identified factors, specific interventions can be developed. On the one hand, children’s fear and pain during the blood collection could be addressed (e.g., distraction, relaxation techniques, pain reduction), and on the other hand, support could be provided to parents before the blood collection (e.g., psychoeducational information with a detailed explanation on the venipuncture process).

## Figures and Tables

**Table 1:** Means (*M*) and standard deviations (*SD*) of main variables by gender, *t*-test for independent samples.

Measure	Girls *M* (*SD*)	Boys *M* (*SD*)	Stat. Comparison of Mean Values
State anxiety child s.r. ^1^	2.41 (1.40)	2.31 (1.40)	*t*(121) = −0.41, *p* = 0.686
State anxiety child p.r. ^2^	3.07 (1.38)	2.79 (1.49)	*t*(141) = −1.14, *p* = 0.258
Trait anxiety child s.r. ^1^	1.64 (0.36)	1.56 (0.44)	*t*(83) = −0.87, *p* = 0.388
Trait anxiety child 0–3 p.r. ^2^	1.66 (0.67)	1.63 (0.71)	*t*(59) = −0.17, *p* = 0.865
Trait anxiety child 4–7 p.r. ^2^	1.61 (0.35)	1.54 (0.37)	*t*(59) = −0.70, *p* = 0.485
Trait anxiety child 8–18 p.r. ^2^	1.64 (0.37)	1.55 (0.32)	*t*(84) = −1.09, *p* = 0.279
Pain child s.r. ^1^	3.34 (3.06)	2.73 (2.81)	*t*(120) = −1.15, *p* = 0.254
Pain child p.r. ^2^	3.97 (2.68)	3.76 (2.57)	*t*(142) = −0.48, *p* = 0.635
State anxiety parents s.r. ^1^	1.74 (0.64)	1.81 (0.80)	*t*(145) = 0.56, *p* = 0.575
Stress SI ^3^ child s.r. ^1^	3.52 (1.13)	2.56 (1.17)	*t*(62) = −3.33, *p* = 0.001
Stress SI ^3^ child p.r. ^2^	3.56 (1.22)	3.58 (1.18)	*t*(119) = −0.09, *p* = 0.931
Stress SI ^3^ parent s.r. ^1^	4.54 (0.69)	4.48 (0.93)	*t*(132) = −0.42, *p* = 0.677
Stress SI ^3^ parent c.r. ^4^	4.47 (0.74)	3.98 (1.24)	*t*(49.21) = −1.97, *p* = 0.055

^1^ s.r. = self-report ^2^ p.r. = parent report ^3^ SI = surgical intervention ^4^ c.r. = children report.

**Table 2:** Means (*M*) and standard deviations (*SD*) of main variables by age group.

Variable	0–3 Years		4–7 Years		8–18 Years	
	Self- Report	Other Report	Self- Report	Other Report	Self- Report	Other Report
State anxiety child (CFS ^1^)	-	3.59 (1.53)	3.21 (1.45)	3.59 (1.34)	1.99 (1.19)	2.48 (1.28)
Pain child (FACES ^2^)	-	5.91 (2.73)	4.84 (3.28)	4.22 (2.57)	2.26 (2.42)	3.17 (2.31)
Trait anxiety child (STAIC-(P)-T ^3^)	-	1.70 (0.70)	-	1.58 (0.38)	1.60 (0.39)	1.60 (0.35)
Stress SI ^4^ child	-	3.39 (1.09)	-	3.72 (1.16)	3.07 (1.24)	3.55 (1.24)
State anxiety parent (CFS ^1^)	-	-	-	2.03 (1.15)	-	1.62 (0.80)
State anxiety parent (SKD ^5^)	2.03 (0.88)	-	1.83 (0.82)	-	1.68 (0.60)	-
Stress SI ^4^ parent	4.86 (0.32)	-	4.62 (0.67)	-	4.38 (0.92)	4.26 (1.01)

^1^ CFS = Children’s Fear Scale. ^2^ FACES = Wong–Baker FACES scale. ^3^ STAIC-(P)-T = Spielberger’s State-Trait Anxiety Inventory for Children—(Parent) – Trait Version, Trait anxiety values were obtained using standardized instruments (STAIC-T and STAIC-P-T) only for children aged 8–18 years. ^4^ SI = surgical intervention. ^5^ SKD = State-Trait Anxiety Inventory. Note that with respect to children’s measurements, other report means parental report, whereas with respect to parent measurements, other report means report by the child. Scales for all variables range from 1 to 5, except the two pain-related variables, which range from 0 to 10.

## Data Availability

The data presented in this study are available on request from the corresponding author.
